# MCT8 Deficiency in Infancy: Opportunities for Early Diagnosis and Screening †

**DOI:** 10.3390/ijns11030066

**Published:** 2025-08-21

**Authors:** Ilja Dubinski, Belana Debor, Sofia Petrova, Katharina A. Schiergens, Heike Weigand, Heinrich Schmidt

**Affiliations:** 1 Division of Paediatric Endocrinology and Diabetology, Dr. von Hauner Children’s Hospital, University Hospital, Ludwig-Maximilians-Universität (LMU), 80539 Munich, Germanykatharina.schiergens@med.uni-muenchen.de (K.A.S.); heinrich.schmidt@med.uni-muenchen.de (H.S.); 2 Centre for Paediatric Endocrinology Zurich (PEZZ), Möhrlistrasse 69, 8006 Zurich, Switzerland; 3 Department of Pediatrics, Klinikum Dritter Orden, 80638 Munich, Germany; sofia.petrova@dritter-orden.de; 4 Division of Paediatric Neurology, Center for Comprehensive Developmental Care (CDeCLMU), Developmental Medicine and Social Paediatrics, Dr. von Hauner Children’s Hospital, University Hospital, Ludwig-Maximilians-Universität (LMU), 80539 Munich, Germany; heike.weigand@med.uni-muenchen.de

**Keywords:** MCT8 deficiency, Allan–Herndon–Dudley syndrome, Tiratricol

## Abstract

Background: Monocarboxylate-transporter-8-(MCT8) deficiency, or Allan–Herndon–Dudley syndrome (AHDS), is a rare X-linked disorder caused by pathogenic variants in the SLC16A2 gene, leading to impaired transport of thyroid hormones, primarily T3 and T4, across cell membranes. The resulting central hypothyroidism and peripheral hyperthyroidism cause neurodevelopmental impairment and thyrotoxicosis. Despite the availability of therapy options, e.g., with triiodothyroacetic acid (TRIAC), diagnosis is often delayed, partly due to normal TSH levels or incomplete genetic panels. MCT8 deficiency is not yet included in newborn-screening programs worldwide. Case Description: We present a case of an infant genetically diagnosed with MCT8 deficiency at 5 months of age after presenting with muscular hypotonia, lack of head control, and developmental delay. Thyroid function testing revealed a normal TSH, low free T4, and significantly elevated free T3 and free T3/T4 ratio. Treatment with TRIAC (Emcitate^®^) was initiated promptly, with close drug monitoring. Despite persistent motor deficits and dystonia, some developmental progress was observed, as well as reduction in hyperthyroidism. Discussion/Conclusions: This case underscores the importance of early free T3 and fT3/fT4 ratio testing in infants with unexplained developmental delay. Broader inclusion of SLC16A2 in genetic panels and consideration of newborn screening could improve early diagnosis and outcomes in this rare but treatable condition.

## 1. Introduction

Monocarboxylate transporter 8 (MCT8) is encoded by the gene SLC16A2, which is localized on Xq13.2. This transporter is necessary for the transmembrane transport of both tri-iodothyronine (T3) and thyroxine (T4). Thyroid hormones play a prominent role in the regulation of metabolic processes as well as in the development and growth of various tissues, first and foremost nerve tissue [1,2]. MCT8 is specific for the thyroid hormones mentioned above and is ubiquitously expressed in numerous tissues of the human body [3,4,5,6]. The consequence of a malfunction of the transporter is a severe neurodevelopmental disorder. The syndrome is also known as Allan–Herndon–Dudley syndrome (AHDS; OMIM No. 300523) or MCT8 deficiency. The prevalence is estimated at 1:70,000 in boys [7]. However, the pathophysiology is characterized by central hypothyroidism (due to the transporter defect) and peripheral hyperthyroidism, up to thyrotoxicosis. In addition, numerous comorbidities can be held responsible for a reduced life expectancy [6,7,8]. The clinical presentation is determined by the severest cognitive and motor developmental disorders as well as associated sequelae (Table 1). Here, the long-term central hypothyroidism and concomitant peripheral hyperthyroidism must be particularly emphasized as the pathophysiological basis [8,9]. The median age at diagnosis is 24 months, whereas the median age at onset of first symptoms is 4 months [7]. The delay between symptom onset and diagnosis has a median duration of 18 months, a diagnostic gap that could potentially be closed by newborn screening. The most frequently reported initial concerns prompting medical evaluation were gross developmental delay, hypotonia, feeding problems, and poor weight gain [7]. MCT8 deficiency is associated with substantially reduced overall survival, with a median life expectancy of only 30 years [7].

Different therapeutic approaches at varying stages of development are in the pipeline [10]. As of 2025, there are solid data that triiodothyroacetic acid (TRIAC) can alleviate certain symptoms, particularly those associated with peripheral hyperthyroidism [7,11,12]. TRIAC (trade name: Emcitate^®^) received a marketing authorization as an orphan drug in February 2025 by the European Medicines Agency (EMA). While TRIAC has shown convincing effects on brain development and neurological outcomes in animal models, comparable evidence in humans remains lacking [13,14], and no therapies are currently available that have been shown to effectively prevent or reverse the neurological deficits associated with the disease.

However, the diagnosis is often delayed and even genetic testing is not always accurate, as the SLC16A2 gene is not always part of the panel [15,16]. The implications in female patients (despite a paradoxically X-linked inheritance) with such a heterozygous mutation have only recently been highlighted [17].

Early treatment appears to be particularly important and should therefore be carried out without delay. Whether this disease could become part of a newborn screening program is the subject of current research [18,19]. Since patients with a developmental delay are not necessarily initially investigated for a thyroid hormone disorder and free T3 is not necessarily always determined, this case report serves to raise awareness of this rare clinical condition and to discuss the potential utility of newborn screening for MCT8 deficiency. In this case report we would like to share our experience that led to a very early diagnosis of an affected boy in infancy.

## 2. Case Description

### 2.1. Diagnosis and Early Treatment Initiation During Infancy

**Figure 1:**
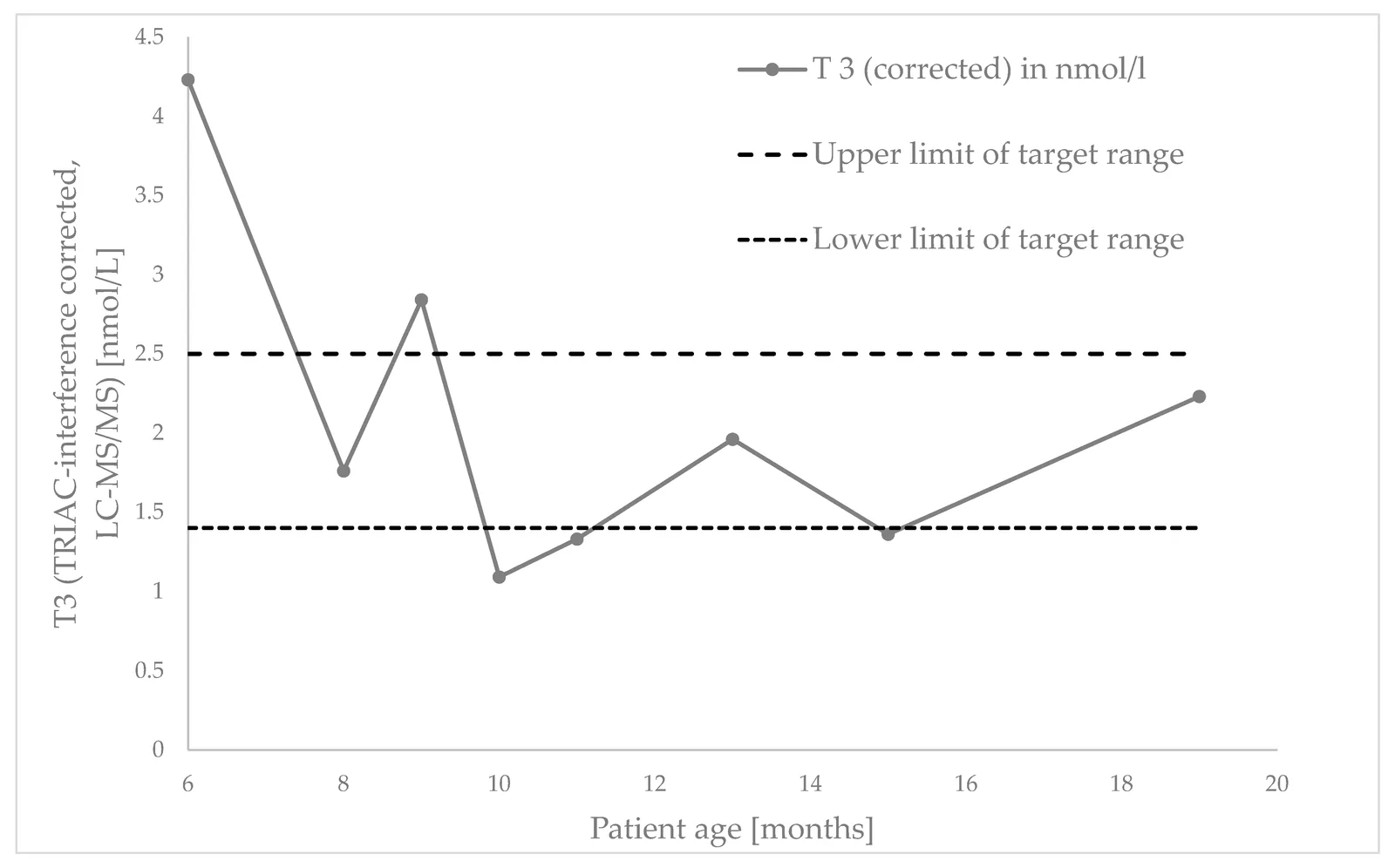
Course of interference-free T3 (LC-MS/MS) as a surrogate marker for treatment response during TRIAC titration.

We would like to put forward a case of an infant who presented with muscular hypotonia, lack of head control, and developmental delay at the age of 5 months. The patient was admitted to hospital for neurological developmental diagnostics due to muscular hypotonia. The pregnancy and birth were unremarkable. In a first laboratory investigation for the etiological classification of the neurological symptoms, a normal TSH with 5.04 µU/mL (reference 0.73–8.35 µU/mL), a reduced free T4 with 0.6 ng/dL (reference 0.9–2.0 ng/dL), and a significantly increased free T3 of 9.7 pg/mL (reference 2.2–5.8 pg/mL) were found. Laboratory testing for both thyroid hormones (free T3 and free T4) is standard practice at our hospital. Except for a somewhat delayed myelination, no morphological abnormalities were found in the MRI of the head. The EEG examination showed a generalized slowing. The clinical examination revealed a lack of head control. A targeted genetic test was carried out with the detection of a pathological mutation in the SLC16A2 gene on the chromosome Xq13.2 (c.474_475del, p.(Glu158AspfdTer35), hemizygous), responsible for MCT8 deficiency. After diagnosis, therapy with TRIAC was started immediately with increasing doses. Treatment was started with 1 dose of 175 micrograms of TRIAC per day. Drug levels and hormone values were monitored at short intervals in close cooperation with Erasmus Medical Centre Rotterdam, The Netherlands. The measurement of (free) T3 under TRIAC therapy is cumbersome in routine laboratories due to interference in most if not all T3 immunoassays [20,21] and must be carried out using LC-MS/MS methods. The course of the corrected free T3 hormone in our patient is shown in Figure 1. The dose was increased gradually to 700 micrograms in the morning, 350 micrograms at midday, and 350 micrograms in the evening. The heterogeneity of the clinical symptoms, some of which are not directly attributable to hyperthyroidism, necessitates multidisciplinary care. Due to myotonic crises, insomnia/restlessness, and feeding disorders/dyspepsia, concomitant pharmacological therapies may also be necessary. Our patient received proton pump inhibitors, melatonin, chloralhydrate, and analgesics (ibuprofen/paracetamol) as clinically required. With some medications that could influence the absorption of Emcitate^®^, special care should be taken and a laboratory assessment of the therapy dosage may be necessary.

### 2.2. Follow-Up at Three Years

The patient, diagnosed in infancy with a confirmed MCT8 deficiency, presents at 3 years (current dosage of TRIAC: 1400 micrograms/day in three doses) with pronounced global hypotonia and axial weakness, combined with intermittent dystonia and fluctuating muscle tone. Head control remains poor, and the child is unable to sit independently. However, he can maintain a relatively stable posture when supported. Dystonic and spastic movement patterns of the limbs and facial musculature occur variably, particularly in response to external stimuli or discomfort. Active locomotion is absent (no crawling or independent sitting), although attempts to roll over are observed. There is evidence of some purposeful hand movements.

Cognitive development, while globally delayed, is encouraging within the context of the syndrome: the child consistently establishes and maintains eye contact, smiles responsively, and appears to comprehend simple interactions. No speech development is present, though occasional vocalizations occur.

Oral feeding is significantly impaired due to orofacial hypotonia and dystonia. The family has so far opted against the placement of a percutaneous endoscopic gastrostomy (PEG) tube. Gastroesophageal reflux is frequent and under gastroenterological management. Growth and nutritional status are compromised, as expected.

Respiratory function is generally sufficient, although there is an increased tendency for secretion retention during infections or episodes of hyperextension. No hospital admissions have been required for respiratory complications. Despite ongoing treatment with TRIAC, the patient remains persistently tachycardic, and initiation of β-blocker therapy is currently under consideration. A reassessment of the therapeutic dosage should also be considered. No epileptic seizures have occurred to date.

The patient has a marked sleep disorder, currently managed with chloralhydrate, occasionally administered twice nightly, with moderate efficacy.

## 3. Discussion/Conclusions

Our case highlights the diagnostic value of early thyroid hormone profiling in infants with developmental delay. With this case report we would like to describe our experience of a very early diagnosis of a patient with MCT8 deficiency with the greatest potential benefit of early treatment. Relying solely on TSH measurements may be insufficient, as TSH values are often within the normal range in MCT8 deficiency (Table 1). Instead, the characteristic constellation—markedly elevated free T3, slightly reduced or normal free T4, and normal or mildly elevated TSH—should prompt further evaluation [10]. Calculation of the free T3/free T4 ratio is a simple, rapid, and widely available diagnostic tool that can support early suspicion of MCT8 deficiency or thyroid hormone resistance syndromes. The use of age-adjusted percentiles for this ratio may facilitate timely recognition and diagnostic confirmation [20]. The typical morphological phenotypic appearances of patients with MCT8 deficiency/AHDS develop over years and are not expedient, especially in the infant phase. Thus, the combination of developmental delay and lack of head control (or even just one symptom) should always lead to a measurement of free T3 hormone (and ideally the calculation of the free T3/T4 ratio). In addition, the SLC16A2 gene should always be included in genetic panel screenings. Correspondingly, our patient exhibited a typical lab profile that led to the definitive genetic diagnosis.

From birth up to approximately 4 months of age, alterations in reverse T3 (rT3) levels and the rT3/T3 ratio serve as important early diagnostic markers, whereas increases in free T3 and changes in the free T3/free T4 ratio generally become apparent only after this period [7,18]. In our patient, reverse T3 measurement was not performed because the free T3 levels and the free T3/free T4 ratio were already found to be pathological. In cases where thyroid hormone values are inconclusive, genetic analysis may offer a diagnostic pathway.

The identified mutation represents a previously unreported frameshift variant, making it a rare genetic alteration. In vitro analyses have shown that MCT8 proteins in mild phenotypes are expressed on the cell membrane and retain the ability to transport T3, whereas frameshift mutations abolish MCT8 expression entirely, resulting in a severe phenotype [22]. Consistent with this, the present case displayed a severe phenotype. Without treatment, progressive neurodevelopmental impairment would be expected, and late initiation of TRIAC therapy is unlikely to reverse established neurological deficits. Therefore, early recognition—prior to irreversible neuronal damage—offers the greatest potential to optimize developmental outcomes, even if some symptoms may only improve slightly. Hypothetically, even prenatal treatment could be considered in the future, although prenatal diagnosis is currently not feasible since pregnancies typically proceed without abnormalities, and cases are almost always diagnosed postnatally.

As highlighted in [21], all commercial total and free T3 immunoassays show significant cross-reactivity with TRIAC. Therefore, for patients receiving TRIAC therapy, monitoring T3 levels using alternative methods that do not rely on immunoassays (e.g., LC-MS/MS) is recommended to avoid misinterpretation of thyroid function tests. For this reason, Figure 1 presents the TRIAC-interference-free T3 values determined by LC-MS/MS.

The potential for newborn screening for MCT8 deficiency should be considered in the context of established screening principles. MCT8 deficiency is a rare X-linked disorder marked by early-onset developmental delay, central nervous system involvement, and peripheral thyrotoxicosis. While current therapies have not been shown to alter the neurological trajectory, they can effectively normalize peripheral thyroid hormone levels [11,12]. Given the severity of the thyrotoxic state and its known long-term consequences, early identification and treatment may provide meaningful somatic benefits, particularly if initiated early. Screening could enable timely diagnosis, improve management of peripheral symptoms, and facilitate family counseling and access to emerging therapies or clinical trials. Concerns about potential harms—such as parental anxiety or healthcare overutilization—must be weighed against these tangible benefits. Furthermore, recent studies [18,19] have demonstrated that thyroid hormone analysis in dried blood spots, especially the combination of low reverse T3 (rT3) and elevated T3/rT3 ratio, may allow for specific and sensitive early detection. While further validation and long-term outcome data are needed, these findings support continued evaluation of MCT8 deficiency as a candidate for inclusion in newborn screening programs.

We would like to formulate the following Take Home Messages from the discussion: 1. Developmental delays should always be the reason for the measurement of free T3 thyroid hormone as part of the investigation. 2. The calculation of the free T3/T4 ratio is widely available, inexpensive, and quick to perform. A ratio of over 0.75 or over the 97th percentile is indicative of MCT8 deficiency [20,23]. 3. In clinically suspected MCT8 deficiency, rT3 (and T3/rT3 ratio) measurement before 4 months may aid early diagnosis, as typical T3 rise occurs later [7,18]. 4. Genetic panel diagnostics for developmental delay or thyroid disorders should always include the SLC16A2 gene. 5. The available therapeutic options should be used early to reduce hyperthyroidism-related symptoms/thyrotoxicosis. The improvement in neurological outcome is still unclear [24]. 6. Inclusion of MCT8 deficiency in newborn screening requires further research, as only a subset of clinical symptoms can currently be addressed therapeutically.

## Figures and Tables

**Table 1:** Clinical phenotype of MCT8 deficiency (summarized in accordance with [7,8,9,10], without claim to completeness) *.

Category	Typical Phenotype
General signs	Severe underweight (71.1%) Feeding problems (71.4%) Mild-to-moderate intellectual disability (100%) Severe delay in motor development (100%) Truncal hypotonia (100%) Lack of head control (75.3%)
Neurological signs	Seizures (with EEG signs) (23.1%) Neurodevelopmental delay (100%) Sleep problems (39.2%) Apneusis (21.9%) Peripheral hypertonia (90.5%) Dystonia (82.6%)/Spasticity (80.3%) Persistent primitive reflexes (91.1%) Delayed myelination (100%) Abnormal cerebral white matter volume (100%) Periventricular WML/Prominent ventricles (100%) Scoliosis (88.2%) Hip luxation (66.7%)
Cardiovascular signs	Elevated systolic blood pressure (53.2%) Premature atrial complexes (75.6%) Tachycardia in rest (31.3%) Sudden death (18.8%) Pulmonary infection (69.1%)
Thyroid function tests	Chronic thyrotoxicosis Serum T3 ↑↑↑ (95.1%) Serum free T4 ↓/⇔ (88.7%) Serum T3/T4 ratio ↑↑↑ (90.5%) Serum TSH ⇔/slightly ↑(88.6%)
Other lab results	Serum sex hormone binding globulin (SHBG) ↑ (88.5%) Serum creatinine ↓/⇔ (27.8%) Serum creatine kinase (CK) ↓/⇔ (96.2%)

* There are many further symptoms in the sense of an “phenotyping of neurodevelopmental features” [7]. The quantification information relates to the published data from [7].

## Data Availability

The original contributions presented in this study are included in the article. Further inquiries can be directed to the corresponding author.

## Abbreviations

The following abbreviations are used in this manuscript:

MCT8	Monocarboxylate transporter 8
AHDS	Allan–Herndon–Dudley syndrome
TRIAC	Triiodothyroacetic acid (Trade name: Emcitate^®^)
